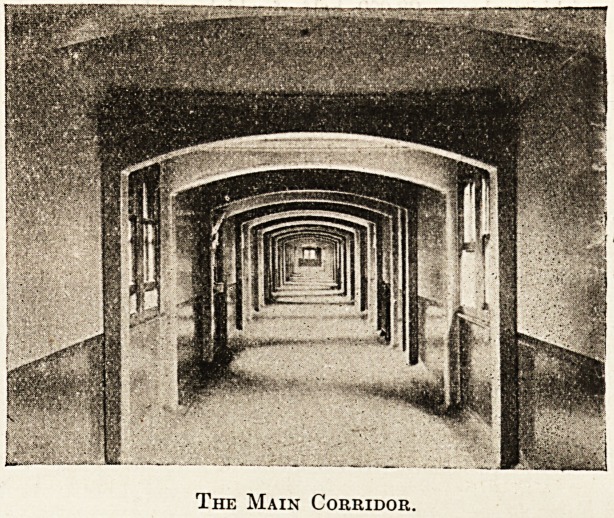# The Evolution of a County Hospital

**Published:** 1912-02-03

**Authors:** 


### THE EVOLUTION OF A COUNTY HOSPITAL

WITH PHOTOGRAPHS TAKEN BY ONE OF THE RESIDENT MEDICAL STAFF.

"Our hospital at Bedford," said Mr. Beauchamp Wadmore, the Secretary, to our Commissioner, who visited the institution a few days ago, "is, in perhaps a unique manner, the work and creation of one man, or rather one family. Mr. Samuel Whitbread, by his will, dated June 24, 1795, bequeathed to his son Samuel and to two of his nephews, £8,000, half of which was to be expended on hospital buildings and the other half on an endowment. He died the following year, but it was not till 1802 that the building was commenced, and ever since that date the office of President has been held by one of the family. In 1802 it was founded by the late Mr. Samuel Whitbread, and not founded merely but endowed, and ever since that date the office of President has been held by his descendants. At this moment Mr. Samuel Whitbread, our present President, the great-grandson of our founder, takes the keenest interest in the hospital, and his son, Mr. Samuel Howard Whitbread, is one of our trustees. To that family and to the respective Dukes of Bedford the hospital owes very much of its success."

The Men's, and Back of Women's, Wards.

"Your site," I said, "is astonishing in its extent."

"Yes," replied Mr. Wadmore, "it has been estimated at eleven acres, and apart from giving us plenty of breathing space, has allowed the present hospital, a new building as you see, to cover the large amount of ground that the E plan necessitates. Some of the grounds are leased as pasture, and a fraction of the rest is enough to provide the hospital with the vegetables that it requires."

"The lime avenue does not lead up to the main entrance?"

### The New Building.

"No; part of it was cut down when the present building was built, that is to say, in 1898. Before that it led up to the old general infirmary, as it was called, a much smaller building than the present one, and of which the hand laundry which you can see at the side of the present building is all that remains. Building the new hospital was a tremendous undertaking. It cost £40,000, towards which both the Duke of Bedford and Mr. Samuel Whitbread gave £5,000. It took ten years before the debt was paid off. The new building, in which I expect you are more interested than in the one which it replaced, made the hospital one of ninety beds, together with an isolation hospital of ten beds in the grounds, which was put up before the town built its own, and which therefore is not at present in use."

"Have you any photographs of the grounds and buildings?"

### The Hospital's Photographer.

"Mr. H. W. Tilling, the house surgeon, is our photographer," said Mr. Wadmore, laughing. "You must ask him. He will take you round the wards." As will be seen, Mr. Tilling generously placed his large stock of photographs at our disposal, from which the following illustrations have been made with his permission. In the course of our walk through the wards which form the three arms of the E, the men's surgical and medical wards forming the eastern or top arm, the women's wards the centre, and the children's single ward the bottom, Mr. Tilling chanced to say that he was not in favour of cultivating any ground between hospital pavilions. He preferred simple lawns to anything else. When we had left the long corridor which runs at right angles to the ward blocks Mr. Wadmore joined us, and we inspected the chapel, which was given and decorated, with a gallery for the domestic staff, and a hydraulic-power organ, by the late Mr. J. Frederick N. Nutter, for many years chairman of the Board of management.

"Our next place," said Mr. Wadmore, "must be the out-patient block."

"Are any improvements wanted here?" I asked, as we entered.

"One emphatically," Mr. Tilling replied, "and that is better accommodation for the $x$-ray department, which, as you will see, is confined to a room which is far too small, and the apparatus of which is not of the latest pattern."

Turning to Mr. Wadmore therefore I inquired what possibility he could hold out of the necessary money being available for the reconstruction which Mr. Tilling wished for.

The Old Lime-Walk.

"I am not in a position to say whether Mr. Tilling's desires will be fulfilled or not. Our financial position at present is this. Last year, that is from 1910, we carried forward £288. This year, I regret to say, that that balance not only has had to be spent, but we are £500

down in addition, as will be made apparent in our coming report."

"To what do you attribute this?"

The Hand-Laundry.

"To a general increase in the cost of commodities. Practically everything has risen in price. But there is a brighter side to the picture. Last year we received promises of £2,000 in legacies, making 1911 an exceptional year. None of that has yet been received. If Mr. Tilling can persuade the Board, no doubt something may be done for his *x*-ray quarters, in the improvement of which the whole medical staff is keenly interested, as Mr. Tilling has already told you. On the other hand, a suggestion has been made for a new laundry. Our present one, as I told you, is a hand laundry, and costs under £200 a year. Personally I do not think any quarrel can be made with its process or its cheap results, though new and more modern fittings are urgently needed. Either the laundry or the *x*-ray department will probably absorb any money there may be to spare. You must not leave the out-patient department without seeing the dispensary, which has been in charge of Mr. F. Panchaud since 1899, who is also our electrician and radiographer. He is quite an institution in himself, for to him is very largely due the success of our Christmas entertainments. On these occasions he is a host in himself, and his songs are renowned annual events to the permanent staff of the hospital."

"On what do you rely for the support of the hospital?"

No Hospital Sunday.

"Partly on our endowed funds, which realising about £1,600 a year, necessitate the raising of over £3,000 every year by annual subscriptions and donations. Our expenditure is usually about £5,000. The working men's contributions are a very important factor in our solvency. Apart from this it is to be noted that we have no Hospital Sunday. There was a Hospital Saturday movement once, but that has been given up, and in its stead we have

The Dispensary, with Mr. Panchaud in Charge.

hospital week undertaken both in town and country by the Hospital Guild, during which house-to-house collections take place, while the churches and chapels are relied on for harvest-festival collections. In a sense, therefore, we have Hospital Sunday under another name. We keep our accounts on the Uniform System."

"You have taken up the secretaryship only recently?"

"Yes, four months ago I succeeded Mr. W. F. Morley, who had held the post since 1901. In asking me just now what I thought of the "E" plan I can give you a professional answer, for I was an architect for twenty-five years before I became attracted to hospital work. I can therefore give my professional and individual praise to Mr. F. P. Adams, who designed our present buildings, and I consider them excellent both in design and plan."

"Are you thinking of any new developments?"

"Well, a modest one, and that is the grouping of the

The Main Corridor.

subscribers' names in the report, not, as hitherto, according to the number of guineas they subscribe, which has sometimes caused difficulty to them in finding their names, but in alphabetical order subject merely to their general division into governors and subscribers."

"One last word as to the effects of the Insurance Act."

"Already it has lost us five or six subscribers; their defection is directly traced to it."

## Figures and Tables

**Figure f1:**
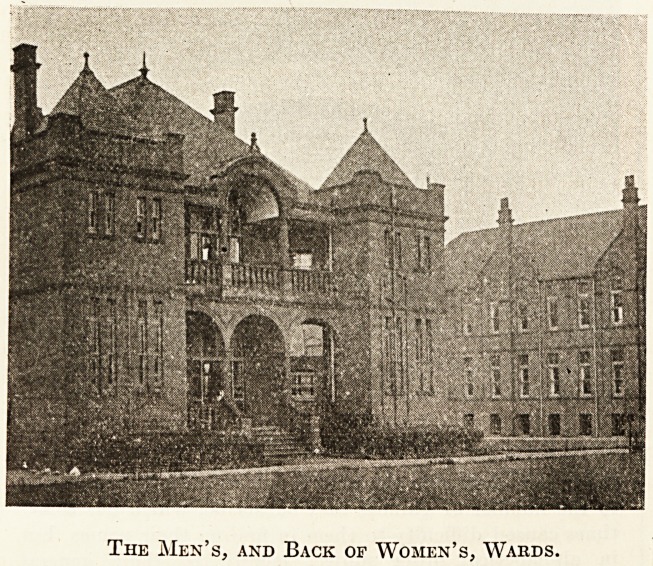


**Figure f2:**
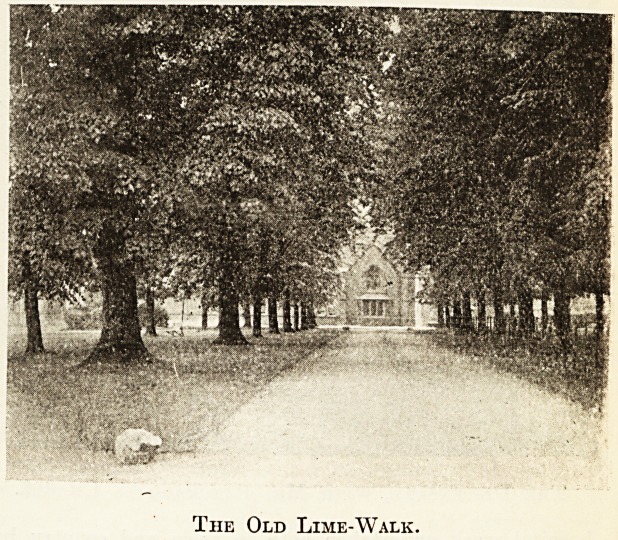


**Figure f3:**
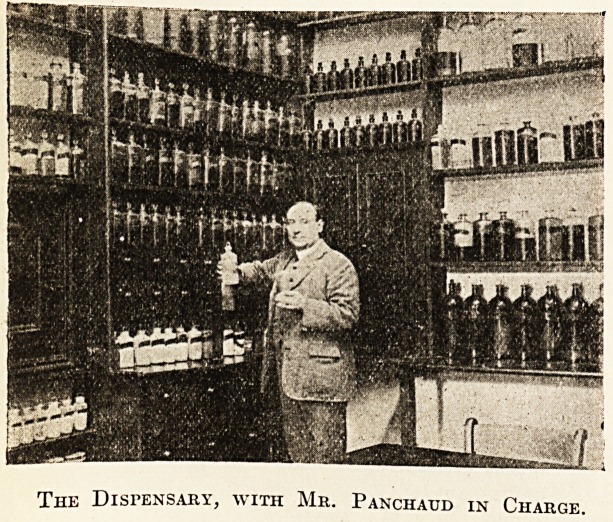


**Figure f4:**